# Comprehensive Analysis of a Japanese Pedigree with Biallelic ACAGG Expansions in *RFC1* Manifesting Motor Neuronopathy with Painful Muscle Cramps

**DOI:** 10.1007/s12311-024-01666-1

**Published:** 2024-02-07

**Authors:** Rumiko Izumi, Hitoshi Warita, Tetsuya Niihori, Yoshihiko Furusawa, Misa Nakano, Yasushi Oya, Kazuhiro Kato, Takuro Shiga, Kensuke Ikeda, Naoki Suzuki, Ichizo Nishino, Yoko Aoki, Masashi Aoki

**Affiliations:** 1 Department of Neurology, Tohoku University Graduate School of Medicine, 1-1 Seiryo-Machi, Aoba-Ku, Sendai, Miyagi, 980-8574 Japan; 2 Department of Medical Genetics, Tohoku University Graduate School of Medicine, Miyagi, Japan; 3 Department of Neurology, National Center Hospital, National Center of Neurology and Psychiatry, Tokyo, Japan; 4 Department of Neurology, Suita Municipal Hospital, Osaka, Japan; 5 Department of Neurology, South Miyagi Medical Center, Miyagi, Japan; 6 Department of Neuromuscular Research, National Institute of Neuroscience, National Center of Neurology and Psychiatry, Tokyo, Japan

**Keywords:** CANVAS, *RFC1*, ACAGG, Muscle cramp, Motor neuronopathy, Telomere

## Abstract

Cerebellar ataxia, neuropathy, and vestibular areflexia syndrome (CANVAS) is an autosomal recessive multisystem neurologic disorder caused by biallelic intronic repeats in *RFC1*. Although the phenotype of CANVAS has been expanding via diagnostic case accumulation, there are scant pedigree analyses to reveal disease penetrance, intergenerational fluctuations in repeat length, or clinical phenomena (including heterozygous carriers). We identified biallelic *RFC1* ACAGG expansions of 1000 ~ repeats in three affected siblings having sensorimotor neuronopathy with spinocerebellar atrophy initially presenting with painful muscle cramps and paroxysmal dry cough. They exhibit almost homogeneous clinical and histopathological features, indicating motor neuronopathy. Over 10 years of follow-up, painful intractable muscle cramps ascended from legs to trunks and hands, followed by amyotrophy and subsequent leg pyramidal signs. The disease course combined with the electrophysical and imagery data suggest initial and prolonged hyperexcitability and the ensuing spinal motor neuron loss, which may progress from the lumbar to the rostral anterior horns and later expand to the corticospinal tract. Genetically, heterozygous ACAGG expansions of similar length were transmitted in unaffected family members of three successive generations, and some of them experienced muscle cramps. Leukocyte telomere length assays revealed comparatively shorter telomeres in affected individuals. This comprehensive pedigree analysis demonstrated a non-anticipating ACAGG transmission and high penetrance of manifestations with a biallelic state, especially motor neuronopathy in which muscle cramps serve as a prodromal and disease progress marker. CANVAS and *RFC1* spectrum disorder should be considered when diagnosing lower dominant motor neuron disease, idiopathic muscle cramps, or neuromuscular hyperexcitability syndromes.

**Supplementary Information:**

The online version contains supplementary material available at 10.1007/s12311-024-01666-1.

## Introduction

Cerebellar ataxia, neuropathy, and vestibular areflexia syndrome (CANVAS) is an autosomal recessive adult-onset, slowly progressive neurologic disorder caused by biallelic expanded 5-bp intronic repeats in the *RFC1.* After the AAGGG was identified as a causative repeat motif [1], the ACAGG motif expansion was found in the Asia–Pacific [2] and Japanese [3, 4] CANVAS cohorts, and it was supposed to be pathogenic. In the past 3 years, the phenotypes associated with biallelic intronic repeats in *RFC1* have been expanded from typical CANVAS to more limited phenotypes involving predominantly or exclusively one of the systems or phenotypes derived from other systems, being summarized as an *RFC1* spectrum disorder [5].

To date, ACAGG expansions (~ 600 to 2000 repeats) have been found mostly in Asian patients, in either biallelic or compound heterozygous states with AAGGG expansions [2–4, 6], sharing a common haplotype of single-nucleotide polymorphisms. The ACAGG-CANVAS was rarely reported in non-Asian regions but was found in European families with a different haplotype origin than Asians [7]. Clinically, patients with biallelic ACAGG expansions have more notable motor neuron manifestations such as fasciculation, muscle atrophy, and weakness than those with AAGGG [4, 8]. Recently, RNA foci were identified in neuronal nuclei of patients with biallelic ACAGG and AAGGG expansions, suggesting that RNA toxicity may be involved in the pathogenesis of CANVAS [9].

Despite advances in phenotyping and pathomechanism, there are not enough pedigree analyses to reveal disease penetrance, intergenerational fluctuations in repeat length, or clinical phenomena involving heterozygous carriers. In this study, we identified biallelic ACAGG expansions in *RFC1* in a Japanese pedigree who had sensorimotor neuronopathy with spinocerebellar atrophy, initially manifesting painful muscle cramps and paroxysmal dry cough and later developing diffuse amyotrophy*.* The manifestations of the three affected siblings closely resembled each other and showed clinical, physiological, histopathological, and genetic homogeneity, which are supposed to be representative features of ACAGG-CANVAS manifesting motor neuronopathy.

The presence of motor neuronopathy in ataxic syndrome has been described in a fraction of autosomal dominant cerebellar ataxias, including spinocerebellar ataxia type 1, type 2 [10, 11], and type 3 (SCA3) [12], and also autosomal recessive cerebellar ataxias such as ataxia with ocular apraxia type 1, type 2, and ataxia telangiectasia [13, 14]. These complications may reflect multisystem neuronal loss and the fundamental function of causative genes like DNA repair [15] or regulating RNA metabolism [16, 17].

The clinical description with over 10 years of observation along with comprehensive analyses in this study will help clarify CANVAS more closely and highlight and detail the aspect of motor neuronopathy.

## Materials and Methods

### Patients

**Fig. 1:**
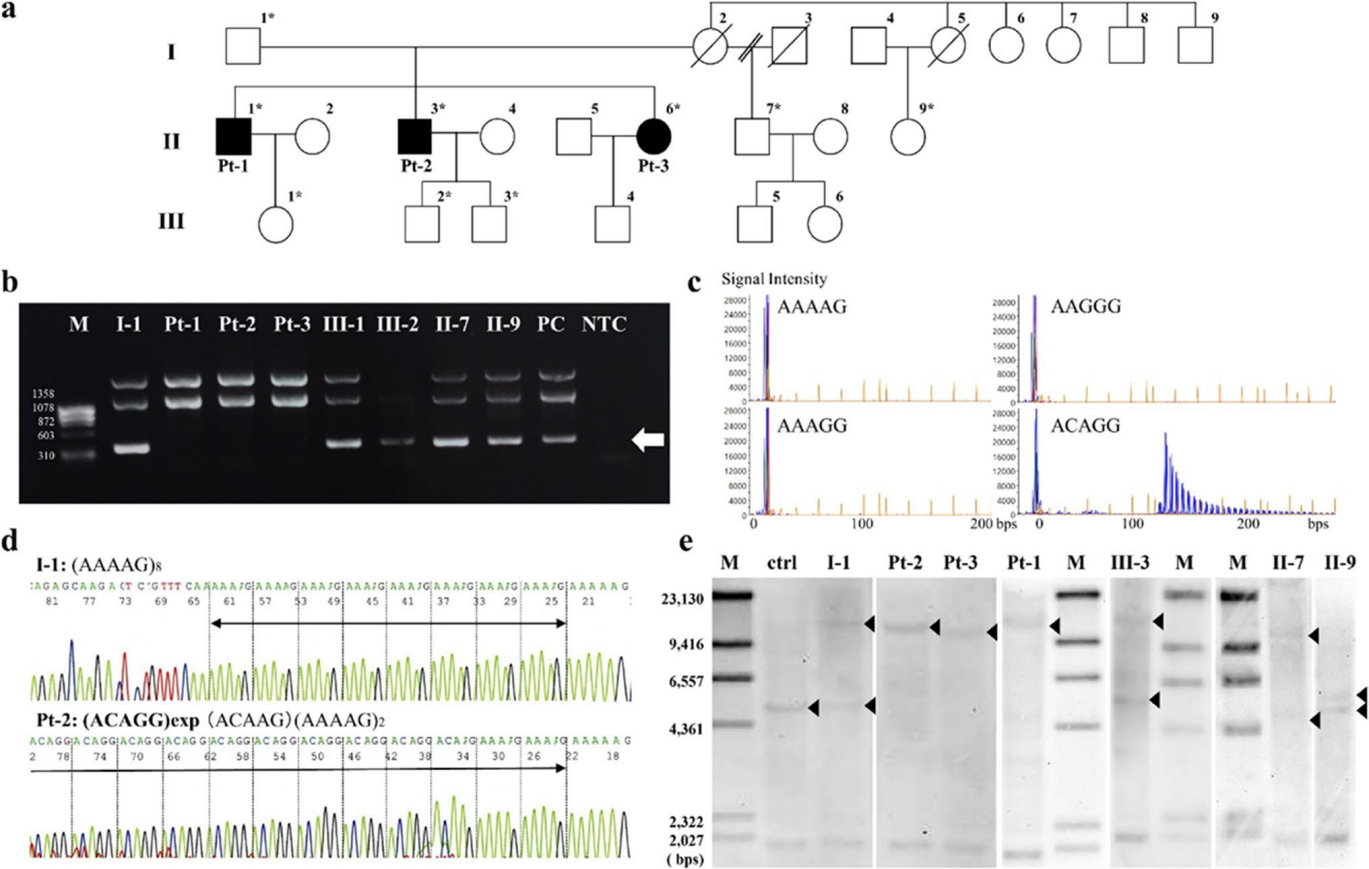
Family pedigree and genetic analysis. The family pedigree is shown (**a**). The family includes three affected individuals with nearly identical manifestations (II-1, II-3, II-6, corresponding to Pt-1, Pt-2, and Pt-3, respectively). Their mother (I-2) complained of muscle cramps and gait disturbances but with insufficient medical information. Non-symptomatic heterozygous carriers of I-1 and II-7 underwent comprehensive examinations that did not show any neurological deficit. Isolated muscle cramps had been recognized in III-1–4 from their childhood to their 30 s. These muscle cramps were essentially non-progressive, and the neurological examination of III-2 and III-3 revealed no sign of the disease. Asterisks indicate individuals whose DNA was used for genetic analyses. The *RFC1* intron 2 short-range flanking PCR demonstrated absent amplification in Pt-1–3, which is estimated to be 348 bps in length (**b**, indicated by an arrow). Per the repeat-primed PCR that was performed, the ladder pattern is only detected via ACAGG-tagged primers in I-1, Pt-1–3, II-7, III-1, and III-2, except in II-9 (**c**). From Sanger-sequencing following nested PCR, I-1 has a referential allele (**d**, shown above), whereas Pt-2 has homozygous expansion of ACAGG motif (**d**, shown below). Per Southern blot analyses (**e**), a wild-type allele fragment supposed to be 5037 bp long is absent in Pt-1–3, and a homozygous single band approximately ranging from 10 to 15 kbps can be seen alternatively. Both expanded and wild-type bands are seen in I-1, II-7, and III-3

The three affected siblings (two males and one female, corresponding to Pt-1 (II-1), Pt-2 (II-3), and Pt-3 (II-6) in Fig. 1a) are of Japanese ancestry and are the only three offspring of non-consanguineous parents. They underwent neurological examinations, electrophysiology, muscle imaging, and biochemical testing. They also underwent nerve and muscle biopsy at the ages of 59 (Pt-1), 57 (Pt-2), and 55 (Pt-3). The Pt-2 and Pt-3 were reevaluated at the ages of 71 and 68, respectively. The clinical profiles of the siblings are summarized in Table 1.

**Table 1:** Clinical features of the siblings with biallelic ACAGG expansion in *RFC1*

Individual/sex	Pt-1/male	Pt-2/male	Pt-3/female	
Age at manifestation				Average age
Paroxysmal dry cough	34	55	50	46.3
Painful muscle cramps	20 s (worsen at 50)	20 s (worsen at 56)	44	50.0
Affected site	Trunk	Calf, abdomen, back	Hand, thigh, calf, abdomen	
Trigger		Cough, exercise	Rest, exercise	
Sensory disturbance	51	54	45	50.0
Gait instability	48	54	54	52.0
Altered taste/dysgeusia	−	54	55	54.5
Blurred vision/oscillopsia	58	55	67	60.0
Sleep apnea	57	58	66	60.3
Age at examination	59	57 (reevaluation at 71)	55 (reevaluation at 68)	
Nystagmus	Gaze-evoked	Gaze-evoked	− (downbeat and gaze-evoked)	
Dysarthria	Slurred speech	− (slurred speech)	− (slurred speech)	
Fasciculation	−	+	−	
Muscle atrophy	Hand, forearm > arm, thigh, calf	Hand, calf (plus forearm)	− (diffuse)	
Muscle weakness	Fingers, gluteus maximum, hamstring, gastrocnemius, toe flexors	Essentially normal	Essentially normal (diffusely MMT4)	
Pyramidal sign	−	− (spasticity in lower limbs)	−	
Postural finger tremor	+	− ( +)	+	
Limb ataxia	+	− ( +)	+	
Romberg sign	+ +	+ +	+ +	
Reflex	Null	Null	Decrease (null)	
Sensory disturbance	All modality	All modality	All modality	
	Distal predominance	Distal predominance	Distal predominance	
Autonomic disturbance	−	ED (plus constipation, urinary incontinence, leg ulcer and chilblain, hypohidrosis)	Constipation (plus urinary incontinence)	
Motor conduction studies (median, ulnar, tibial)	Reduced CMAPs, normal velocity	WNL (small CMAPs, normal velocity)	WNL	
F-waves occurrence (%, right/left)				
Median nerve	15/60	31/44 (19 / 31)	43/N.E	
Ulnar nerve	45/40	44/63 (38 / 25)	65/N.E	
Tibial nerve	75/55	100/75	55/75	
Sensory conduction studies (median, ulnar, sural)	Not evoked	Not evoked	Not evoked	
nEMG				
Spontaneous activity	fibrillation, PSW	fasciculation, CRD (fibrillation, PSW)	− (fibrillation, PSW, myotonic discharge)	
Interference pattern	neurogenic	WNL (neurogenic)	neurogenic	
Number of myelinated nerve fibers ( /mm^2^)	2093	1420	2253	
Muscle pathology	Moderate group atrophy Fiber-type grouping	Small group atrophy Fiber-type grouping	Small group atrophy Fiber-type grouping	
Serum CK (IU/L)	458	600 (193)	150	
Atrophy onMRI				
Cerebellum	+	+	+	
Brainstem	Medulla	Medulla	Medulla	
Spinal cord	Cervical-thoracic	Cervical-thoracic	Cervical (diffuse)	

Abbreviations: *CK*, creatine kinase; *CMAP*, compound muscle action potential; *CRD*, complex repetitive discharge; *ED*, erectile dysfunction; *MMT*, manual muscle testing; *MRI*, magnetic resonance imaging; *N.E.*, not evaluated; *nEMG*, needle electromyography; *PSW*, positive sharp waves; *WNL*, within normal limits; − , absent; + , present

### Muscle and Nerve Histopathology and Immunohistochemistry

Sural nerve biopsy was done in all three patients. Simultaneously, the peroneus brevis (Pt-1, Pt-3) or biceps brachii (Pt-2) muscles were biopsied. Thereafter, the biopsied skeletal muscles were rapidly frozen with isopentane cooled with liquid nitrogen and serially sectioned at 10 µm. The frozen sections were stained per standard procedures, and immunofluorescence staining was performed as previously described [18]. The primary and secondary antibodies used for this procedure are listed in Supplementary Table e-1. Biopsied sural nerves were fixed in 2.5% glutaraldehyde, post-fixed with 1% OsO_4_, embedded in epoxy resin, and subjected to light and electron microscopies per standard procedures.

### Genetic Analysis

All three patients and some of their family members of I-1, II-7, II-9, III-1, III-2, and III-3 gave their consent for the genetic analysis to be performed. Genomic DNA from peripheral blood leucocytes was extracted by standard methods, except for III-2 who had previously undergone bone marrow transplantation. His inherent DNA was extracted from nails using ISOHAIR (NIPPON GENE, Tokyo, Japan).

Whole-exome sequencing was performed on Pt-1–3, I-1, II-7, II-9, and III-1 as previously described [19]. To screen the *RFC1* expansions, short-range flanking PCR and repeat-primed-PCR (RP-PCR) of the AAAAG, AAAGG, AAGGG, and ACAGG repeat sequence were performed as previously reported [1] using Phusion Flash High-Fidelity PCR Master Mix (Thermo Fisher Scientific, MA, USA). In nested PCR analyses, products of the first long amplification reaction were used as the template for the second PCR analysis. PCR products were further analyzed using Sanger sequencing. The PCR conditions and primer pairs are shown in Supplementary Table e-1.

For Southern blotting, digoxigenin (DIG)-labeled probes were prepared using the PCR DIG Probe Synthesis Kit (Roche Applied Science, Penzberg, Germany). A gDNA fragment of a purified PCR product was used as a template. Primer pairs used for PCR amplification of gDNA fragments, the DIG-labeled probe, and the PCR conditions are shown in Supplementary Table e-1. Furthermore, 5 µg of gDNA was digested for 15 min with 50 U of EcoRI-HF (BioLabs, MA, USA) and purified before electrophoresis. Samples and DIG-labeled DNA molecular weight markers II (Roche Diagnostics, Basel, Switzerland) were electrophoresed on 0.8% agarose gel until DNA bands were separated (2–3 h). The transfer of DNA to the membrane to detect the hybridized probe DNA was undertaken essentially as recommended in the DIG application manual (Roche Diagnostics). After pre-hybridization at 45 °C for 3 h, hybridization was allowed to proceed at 45 °C overnight. Approximately 10 μl of PCR products containing the labeled oligonucleotide probe per milliliter of hybridization solution was used for hybridization.

The LTL assay was performed for Pt-1–3, II-7, and II-9, who are of the same generation and were aged 58–69 years when they provided blood samples (1.5 μg of gDNA), by using the Telo *TAGGG* Telomere Length Assay kit (Roche Applied Science) per the manufacturer’s instructions. The assay was performed twice for each sample, and the mean LTL values were obtained.

## Result

### Case Description

#### Disease History

Painful muscle cramps, which had been present as occasional nocturnal muscle cramps restricted to the calves since the patients were in their 20 s (Pt-1 and Pt-2), apparently increased from the age of 50 (Pt-1) and 56 (Pt-2), expanding to the trunk. Pt-3 also experienced painful muscle cramps in the calves and trunk around the age of 44. These painful muscle cramps were easily and frequently evoked by voluntary movements or the below-mentioned cough attacks. The symptoms failed to respond to Shao-Yao-Gan-Cao-Tang; antiepileptic drugs such as clonazepam, carbamazepine, and lamotrigine; or muscle relaxants. The age of onset of chronic paroxysmal dry cough ranged from 34 to 55 years. The cough could arise at any time even when the patient was asleep, either without any specific trigger or with a trigger such as the deglutition of hot food and speech. Such cough attacks gradually worsened in frequency and severity in all cases and often provoked inspiratory dyspnea. Symptomatic treatments using asthmatic, antibiotics, and the above-mentioned antiepileptic drugs were essentially ineffective, except that codeine phosphate partially alleviated the symptoms. Gait instability had progressed since the age of 48 (Pt-1) or 54 (Pt-2 and Pt-3). Pt-2 required a walking aid at 67 and a wheelchair at 68 years of age. Other shared symptoms among the siblings included clumsiness and hypoesthesia of the toes and the fingertips, altered taste or dysgeusia, blurred vision, and sleep apnea. The details are presented in Table 1.

#### Neurological Examination

The siblings were of medium build and physically normal. They maintained normal cognitive and neuropsychiatric function at the ages of 59 (Pt-1), 71 (Pt-2) and 68 (Pt-3). In the cranial nerve examinations, non-sustained horizontal gaze-evoked nystagmus with double vision was observed in Pt-1 and Pt-2. Downbeat and gaze-evoked nystagmus later occurred in Pt-3, and it was found to be of central rather than vestibular origin via electrooculography and optokinetic testing. Speech was found to be slurred or explosive in all cases. There was no auditory impairment or tinnitus in any of the patients, nor did any of them have tongue fasciculation or atrophy. Distal-dominant muscle atrophy was seen initially in the gastrocnemius (GC), thenar, dorsal interossei, and hypothenar muscles, and it was later observed proximally. Pt-2 manifested fasciculations in his four extremities. Deep tendon reflexes were generally attenuated or absent, and there were no pathological reflexes. Lower limb spasticity was observed for the first time at the age of 71 in Pt-2. Limb coordination was mildly disturbed. Although glove-and-stocking-type sensory disturbance was detected in all modalities, the deep sensation impairment was more severe involving the truncus. The patient’s stance was unsteady with a positive Romberg’s sign, and the gait was typically ataxic, suggesting predominant sensory rather than cerebellar involvement. Postural finger tremor was observed in Pt-1 and Pt-3 during the first evaluation, whereas jerky irregular fine finger tremors resembling minipolymyoclonus occurred later in Pt-2. The autonomic nerve system dysfunction in Pt-2 initially manifested as erectile dysfunction, constipation, and urinary incontinence; however, it later progressed to hypohidrosis and leg vascular insufficiency leading to ulcers and chilblains.

#### Laboratory, Radiology, and Electrophysiology

The serum creatine kinase levels were either normal or mildly elevated in all cases (Table 1). Serum albumin, vitamin E, and α-fetoprotein levels were essentially within normal ranges. The cerebrospinal fluid profile was normal. Respiratory functions were also normal. Videofluoroscopic swallowing examinations conducted in Pt-1 and Pt-2 showed normal pharyngeal function during the first evaluation. However, a reevaluation in Pt-2 at the age of 71 revealed decreased tongue motor dexterity without weakness and reduced cough reflex due to laryngeal hypoperception.

Cranial magnetic resonance imaging (MRI) revealed moderate diffuse cerebellar atrophy in all cases (Supplementary Fig. e-1a, b). Atrophy of the medulla oblongata was identified as well. Spinal MRI detected consecutive spinal cord atrophy in the dorsoventral direction, which was obvious from the cervical level to the thoracic level (Supplementary Fig. e-1c). There were no abnormal intraspinal signals. The spinocerebellar atrophy was progressive, which was shown by longitudinal MR imaging in Pt-2, whereas the cerebral volume has been maintained at a normal level.

**Fig. 2:**
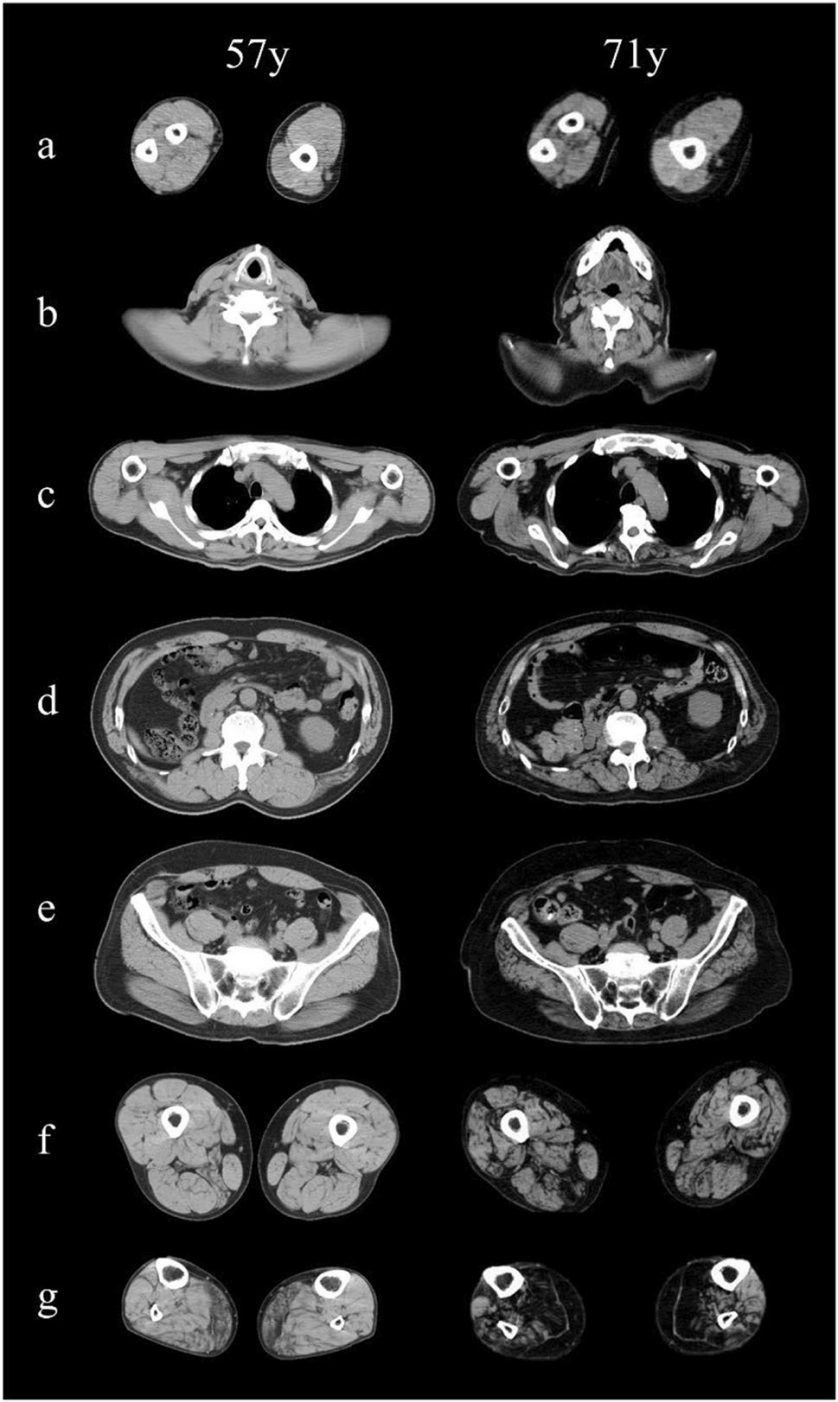
Muscle computed tomography. Muscle computed tomography was evaluated twice in Pt-II at the ages of 57 and 71. The images show slices of the forearm and arm (**a**, left and right), neck (**b**), thorax (**c**), abdomen (**d**), pelvis (**e**), thigh (**f**), and distal legs (**g**). Symmetrical fatty changes in the medial head and to a lesser extent the lateral head of the gastrocnemius are present at 57 years (**g**, left panel), whereas pathological muscle atrophy cannot be identified in other levels. At 71 years, all lower leg muscles were severely fatty-replaced, relatively sparing the tibialis anterior and extensor digitorum longus muscles (**g**, right panel). The thigh exhibited moderate and diffuse atrophy; however, the hamstring muscles are predominantly affected (**f**, right panel). In the truncus, paraspinal muscles are fatty-degenerated continuously from the cervical level to the lumbar level. In the upper extremities, diffuse muscle atrophy is more prominent in the forearms than in the arms (**a**, right panel)

Muscle computed tomography revealed symmetrical fatty changes in the medial head of the GC muscles (Pt-1 and Pt-2) and, to a lesser degree, the lateral head of the GC and soleus muscles. During reevaluation in Pt-2 at 71 years of age, all lower leg muscles were fatty-replaced, relatively sparing the tibialis anterior muscles (Fig. 2). In the forearms, diffuse muscle atrophy was also present. In other sequences, diffuse and symmetric muscle atrophy and fatty changes were seen in proximal limbs and paraspinal muscles.

**Fig. 3:**
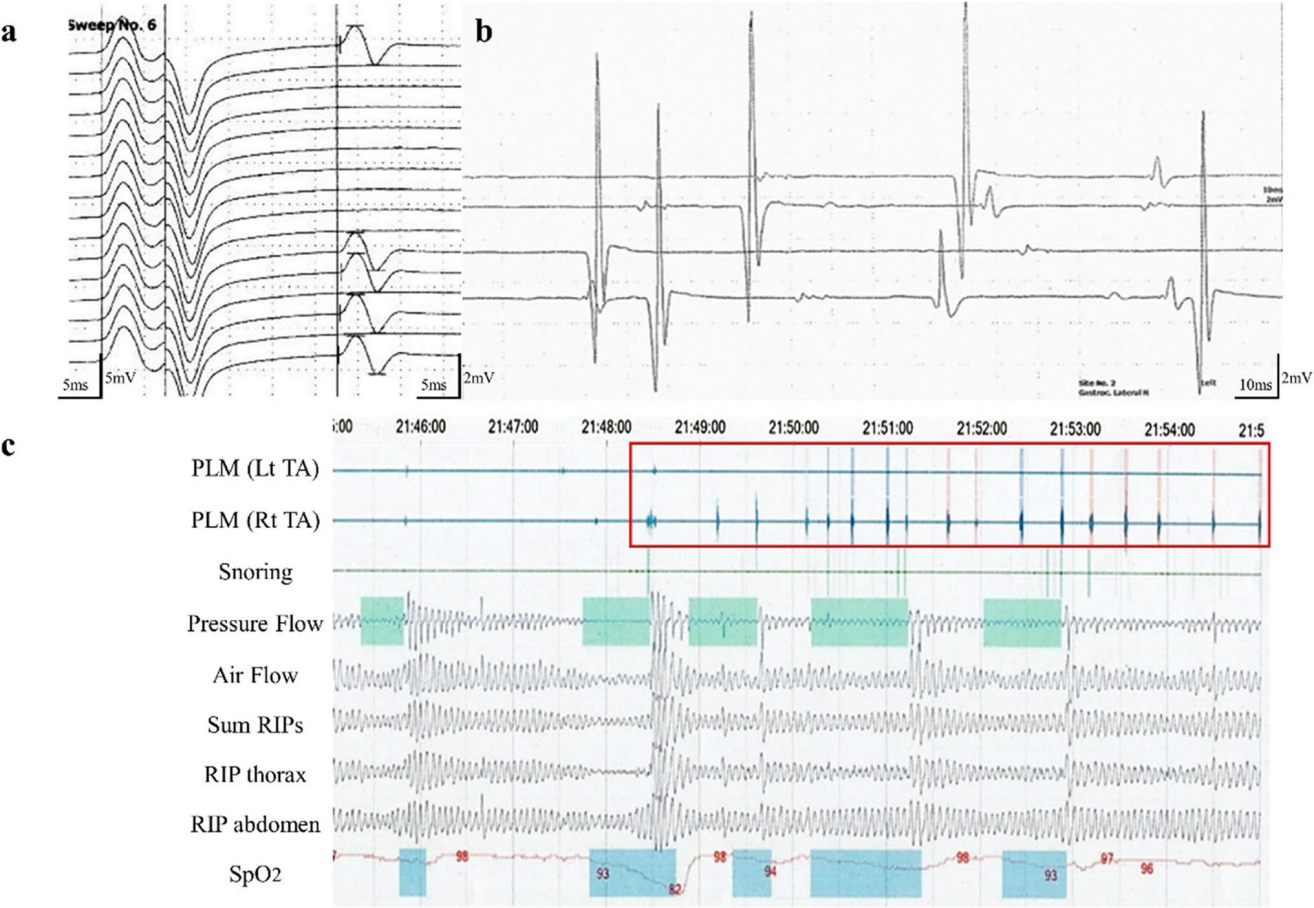
Electrophysiology. F-waves induced by left median nerve stimulation in Pt-2 at the age of 71 are shown (**a**). Whereas the amplitude of the CMAP and motor conduction is maintained at the normal level, the occurrence of F-waves is reduced to 31% occupied by repeater F-waves. The F/M amplitude ratio is increased to 0.4. The nEMG performed on the left gastrocnemius in Pt-3 is shown (**b**). Motor units of simple forms as large as 14 mV are recruited by voluntary contraction. The polysomnography of Pt-3 reveals frequent periodic limb movements (PLMs) that are monitored via both tibialis anteriors (TAs), which frequently occurred early during sleep (**c**) and occurred a total of 219 times during non-rapid-eye-movement sleep. The apnea–hypopnea index, lowest SpO_2_, and maximum apnea duration were 6.7/h, 82%, and 44 s, respectively, indicating mild sleep apnea. RIP: respiratory inductance plethysmography

Electrophysical studies were performed on the three siblings. Motor conduction studies (MCSs) of the median, ulnar, and tibial nerves were essentially normal. F-waves showed normal latencies; however, the occurrence of F-waves variably decreased: 15–60% in the median, 40–65% in the ulnar, and 55–100% in the tibial nerves (Table 1). In addition, MCS of the median and ulnar nerves in Pt-2 at 71 years revealed a further reduction in F-wave occurrence and the full occupancy of high-amplitude repeater F-waves, regardless of normally maintained compound muscle action potentials (CMAPs) (Table 1, Fig. 3a). Sensory nerve action potentials (SNAPs) were not evoked in any of the median, the ulnar, and the sural nerve of Pt-1–3. Short latency somatosensory evoked potentials (SSEP) stimulated in the post-tibial nerves were not evoked from the peripheral in any of the patients. Needle electromyography (nEMG) revealed a chronic neurogenic pattern in the three patients with distal predominancy. Fibrillation potentials and positive sharp waves (PSWs) were detected primarily on the distal limb muscles in Pt-1, and fasciculation potentials or complex repetitive discharges were observed diffusely in Pt-2. Although the initial nEMG in Pt-3 did not record apparent spontaneous activity, the second survey revealed increments in the insertion activity, fibrillation potentials, PSWs, and myotonic discharge. Motor unit potentials were as large as 14 mV in the GC muscle and 15 mV in the quadriceps muscle (Fig. 3b). These nEMG results were consistent with an extremely chronic and still progressing denervation process.

Polysomnography performed in Pt-3 at the age of 68 revealed mild sleep apnea and frequent periodic limb movements (PLM), which frequently occurred early in sleep and caused nocturnal awakening (Fig. 3c).

#### Muscle and Nerve Histopathology and Immunohistochemistry

The affected siblings underwent sural nerve biopsies. The overall myelinated fiber density of the sural nerve was remarkably reduced to 1420–2253/mm^2^ in all of them (normal range 6000–10000/mm^2^). Myelinated large fibers almost disappeared, while small fibers were comparatively preserved (Supplementary Fig. e-2a–c). Myelin ovoid and rare regenerating clusters were rarely found. The myelin structures in the remaining fibers appeared normal. No obvious findings of demyelination or typical onion bulb formation were present. A decrease in the number of small unmyelinated nerve fibers was not apparent under both light and electron microscopies (Supplementary Fig. e-2d).

**Fig. 4:**
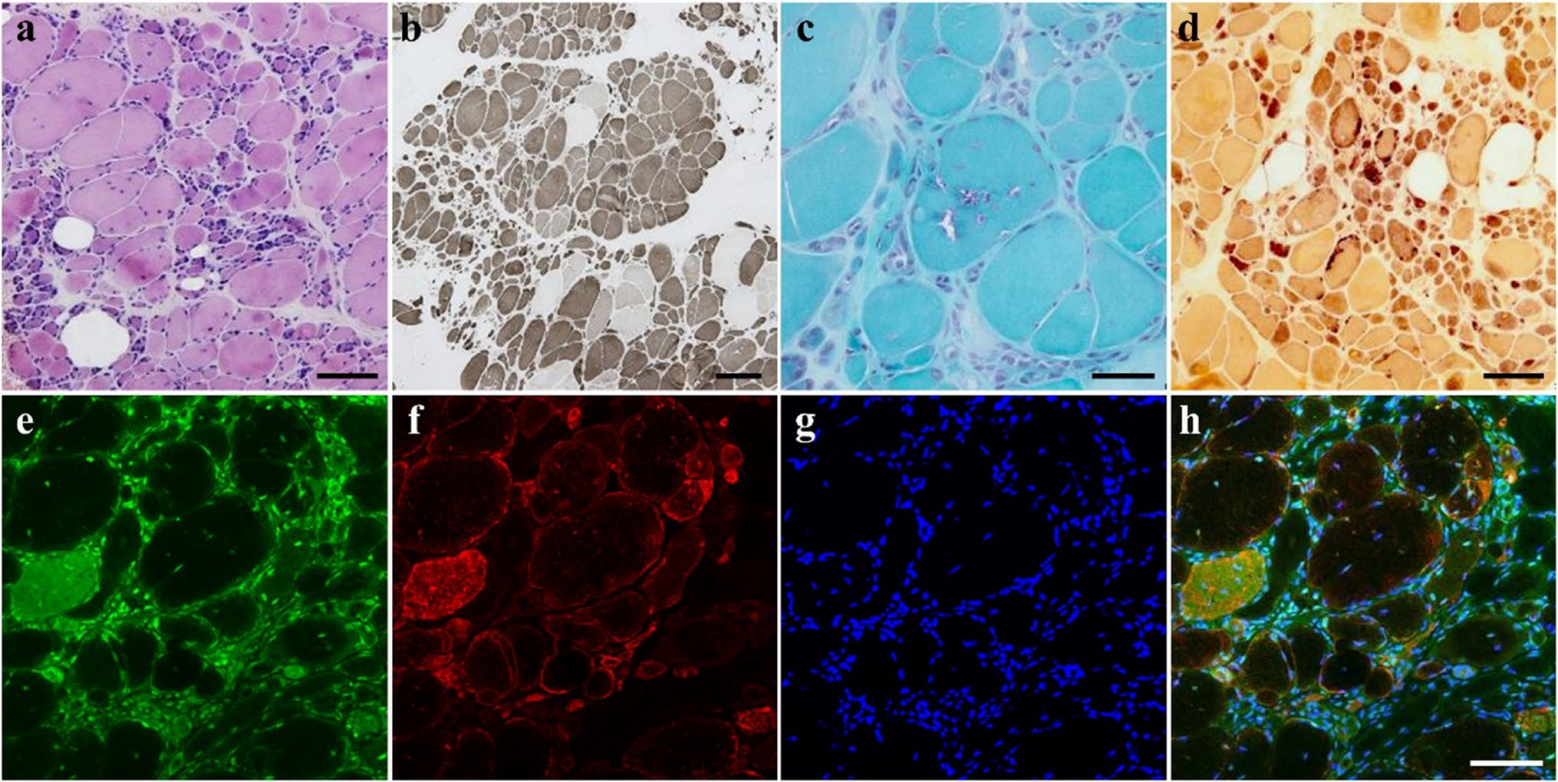
Pathology of muscle biopsy. Hematoxylin–eosin (**a**), ATPase (pH 10.6) (**b**), modified Gomori trichrome (**c**), and nonspecific esterase (**d**) staining of the peroneus brevis muscle sections biopsied from Pt-1 indicate neurogenic changes such as grouped atrophy, pyknotic nuclear clamps, and fiber type grouping (**a**, **b**). Secondary myopathic changes such as endomysial fibrosis, increased fiber size variation, internal nuclei, and rimmed vacuoles are also observed (**c**). Denervated fibers are darkly highlighted (**d**). p62/SQSTM1 positive aggregation was not present in these fibers (data not shown). Per immunofluorescence staining performed in the same specimen, RFC1 (**e**), the neural cell adhesion molecule (N-CAM), a denervation marker (**f**), and Hoechst (**g**) are co-stained and merged (**h**). RFC1 is essentially retained in the myonuclei and is also present in the cytoplasms of atrophic or denervated myofibers. Bar = 100 µm (**a**, **d**, **h**), 200 µm (**b**), 50 µm (**c**)

Biopsied muscle pathology was fundamentally neurogenic, including grouped atrophy and fiber-type grouping. The biopsy of the peroneus brevis muscle in Pt-1 revealed the most obvious chronic neurogenic change and coexisting myopathic changes that included endomysial fibrosis, fiber size variation, internal nuclei, and several rimmed vacuoles scattered in the remaining myofibers (Fig. 4a–d). In immunofluorescence staining, RFC1 was essentially retained in the myonuclei and was also present in the cytoplasms of denervated myofibers (Fig. 4e–h).

#### Genetic Analysis

In whole-exome sequencing, an average of 85.1% (81.58–90.00%) of the overall targeted regions had at least 50-fold coverage. Within the sequenced region, known causative mutations of hereditary neuromuscular diseases listed in the Human Gene Mutation Database (BIOBASE, Waltham, MA) or rare *RFC1* variants were not detected.

In flanking PCR encompassing CANVAS-related regions in *RFC1*, the fragment that was supposed to be 348 bps long if it contained the referential (AAAAG)_11_ sequence was not amplified in Pt-1–3 although it was amplified in other family members (Fig. 1b). The ladder pattern was only detected via ACAGG-tagged primers in I-1, Pt-1–3, II-7, III-1, and III-2 by RP-PCR (Fig. 1c), but it was not detected in II-9. Sanger sequencing using nested PCR products from Pt-2 as a template revealed biallelic ACAGG expansions sequenced from both sides (Fig. 1d). Southern blot analyses showed a wild-type allele fragment supposed to be 5037 bp long was absent in Pt-1–3, while a homozygous single band approximately ranging from 10 to 15 kbps was detected (Fig. 1e). Both expanded and wild-type bands were observed in I-1, II-7, and III-3. These results indicate that Pt-1–3 harbor biallelic ACAGG expansions estimated as 1000 to 2000 repeats, and their father, cousin, and offspring had the expanded ACAGG alleles of the same length as a heterozygous state.

Per the LTL assay among family members of the same generation, the estimated mean LTL was 6.45 kb (Pt-1), 6.54 kb (Pt-2), 7.18 kb (Pt-3), 7.12 kb (II-7), and 7.37 kb (II-9) (Supplementary Table e-2).

## Discussion

In this study, we report a distinct family of patients having CANVAS with biallelic ACAGG expansions. In addition to the core diagnostic features of sensory neuronopathy, cerebellar atrophy, and chronic and intractable paroxysmal dry cough as CANVAS, the patients experienced multisystemic symptoms including dysautonomia and motor neuron manifestations.

The presence of fasciculations, distal-predominant amyotrophy which later became generalized amyotrophy, grouped myofiber atrophy on biopsy, and neurogenic changes on nEMG thoroughly indicate lower motor neuron dysfunction. Motor neuron involvement in CANVAS has been mentioned in previous studies [20, 21] and was reported to be more notable in patients with ACAGG expansions than in those with AAGGG [4, 8]. Nevertheless, the detailed phenotype and natural course as motor neuron disease (MND) and the causative loci have not been fully defined for now.

From electrophysiology, F-wave occurrence had been sequentially decreased to the full occupancy of large repeater F-waves, despite CMAPs being essentially maintained over ten years from first onset. nEMG revealed various abundant spontaneous discharges upon chronic neurogenic changes. These findings indicate a decreased number of spinal motor units and hyperexcitability in the remaining spinal motor neurons, which is consistent with motor neuronopathy. This is supported by the presence of progressive spinal cord atrophy in the cases presented here and the preceding pathological studies reporting the reduction of motor neurons or the axonal swelling of the motor synapse in the spinal anterior horn [20, 22].

A refractory painful muscle cramp is the earlier and most prominent clinical feature shared with affected siblings. Muscle cramps have been reported in a fraction of patients with AAGGG-CANVAS [23, 24], and also in a patient with ACAGG-CANVAS who had experienced severe leg cramps prior to diagnosis [2]. In the family we studied, nocturnal leg cramps gradually expanded to the trunk and hands with increased severity. Subsequently, distal-predominant muscle atrophy progressed over 10 years to become generalized. Eventually, leg spasticity emerged 15 years after the onset of cramps. Supposing muscle cramps are caused by increased lower motor neuronal excitability as is the origin of fasciculation, this time series suggests the symmetrical degeneration of anterior horns that slowly progresses from the lumbar to the rostral spine and the morbidity of the corticospinal tract in a later stage. Whereas this systemic motor involvement is shared with amyotrophic lateral sclerosis (ALS), more severe motor neuron excitability that leads to muscle cramps and milder motor unit loss that in turn causes muscle atrophy and weakness may hint at the distinctive effect on the motor neuron in CANVAS.

The detailed and unique pathophysiology of muscle cramps in CANVAS is unknown; however, muscle cramps as a primary phenomenon reflecting degenerating anterior horn cells are well documented in SCA3 [25] and ALS [26]. In these diseases, muscle cramps are supposed to be caused by motor axonal excitability, which may derive from aberrant ionic conductances, possibly associated with axonal regeneration or collateral sprouting [27, 28]. Together with clinical data in our family, these indicate that similar axonal excitability might underlie CANVAS, and muscle cramps are supposed to be a progress indicator of motor neuronopathy. In addition, the minipolymyoclonus in Pt-2 and the PLM in Pt-3 became apparent 10 years after the onset of the disease. Minipolymyoclonus is frequently observed in MND, and it is derived from fasciculation [29]. Nocturnal PLM is a common manifestation that can be present in the general population, and it can be associated with a variety of neurological disorders and is also hypothesized to result from enhanced spinal cord excitability [30]. Hence, these manifestations are notable as they might be part of the clinical spectrum of motor manifestations of CANVAS.

It is noteworthy that heterozygous repeat carriers who are offspring of patients have been presenting isolated and non-expanding muscle cramps. Two of them underwent medical examinations in their 30 s that did not reveal any CANVAS-related neurological deficits. Regarding other members with muscle cramps, there is inadequate medical information. Thus, additional follow-up and case accumulation are necessary to conclude whether isolated muscle cramps may represent prodromal or milder phenotypes of motor neuronopathy in heterozygous carriers.

Genetic analyses revealed the detailed distribution of repeat sequences among family members. In three successive generations that include heterozygous carriers and homozygous patients, the lengths of ACAGG expansions remained around 1000–2,000 repeats and did not demonstrate any apparent intergenerational or somatic repeat instability. In previous studies on ACAGG-CANVAS mostly from Asian patients, the repeated number of ACAGG motifs ranges from 600 to 2000 [2–4, 6], sharing a common haplotype of single-nucleotide polymorphisms. These, along with our cases, indicate that expanded ACAGG alleles may originate from a common Asian founder and have been inherited in a relatively stable state. The relationship between repeat lengths and clinical phenotypes has not been established so far and requires further case accumulation.

The pathogenic mechanism underlying biallelic *RFC1* expansion is still unknown. *RFC1* encodes the large subunit of replication factor C, a 5-subunit DNA polymerase accessory protein, which is a DNA-dependent ATPase required for eukaryotic DNA replication and repair [15]. RFC1 may also have a role in telomere stability, acting as the initial sensor of telomere damage [31], and its mutants show decreased telomere lengths and reduced telomerase expression [32]. In Friedreich ataxia, a similar autosomal recessive disorder caused by repeat expansion in *FXN*, LTL shortening, has been reported in patient leukocytes [33, 34]. To determine the effect of *RFC1* expansion on telomere length, we performed LTL assays, which have not yet been adapted for CANVAS, on patients and unaffected family members of similar age. The mean LTL values were found to be within mean ± SD of those in a healthy population [35]; however, it is of note that the LTL values in Pt-1 and Pt-2 tended to be shorter than that in a heterozygous carrier (II-7) or non-carrier (II-9). Further analyses in a larger number of patients are necessary to determine whether shorter telomere-mediated genomic instability might be associated with the pathogenesis of the condition and whether LTL could be a possible disease biomarker of CANVAS.

## Conclusion

In conclusion, we report a representative family with ACAGG motifs, demonstrating progressive motor neuronopathy as a convincing clinical aspect of CANVAS. Genetically, ACAGG motifs were homogeneously transmitted, and we did not find an anticipation phenomenon. Clinically, painful muscle cramps are the hallmark of motor neuronopathy and could serve as a prodromal and disease progress marker. Accordingly, CANVAS or *RFC1* spectrum disorder should be considered when diagnosing lower dominant MND, idiopathic muscle cramps, or neuromuscular hyperexcitability syndromes. The detailed pathomechanism upon motor neuron alteration, a manifestation of heterozygous carriers, and the implication of LTL are yet to be elucidated and require further basic research and case studies.

### Supplementary Information

Below is the link to the electronic supplementary material.

Supplementary file1 (PDF 128 KB)

Supplementary file2 (PDF 89 KB)

## Data Availability

All data generated or analyzed during this study are included in this published article and its supplementary materials.
