# Supplementary material for: Comprehensive Analysis of a Japanese Pedigree with Biallelic ACAGG Expansions in RFC1 Manifesting Motor Neuronopathy with Painful Muscle Cramps
Source: Cerebellum. 2024 Feb 7;23(4):1498–508. doi: 10.1007/s12311-024-01666-1 (PMC11269323; doi:10.1007/s12311-024-01666-1)
Supplement: Supplementary file 1 — Supplementary file1 (PDF 128 KB) [file 12311_2024_1666_MOESM1_ESM.pdf]

Supplementary Figure e-1. Cranial and spinal cord magnetic resonance imaging

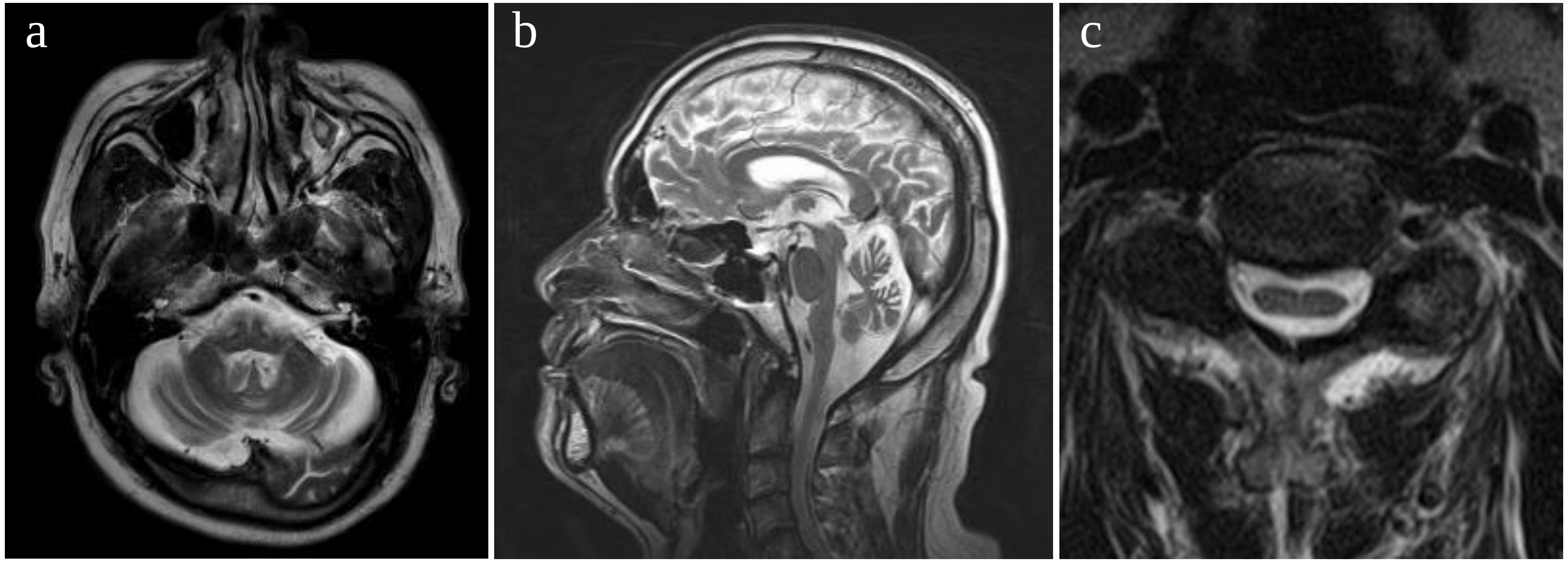

T2-weighted axial images of the cranial (a), cervical (b), and spinal cord (c) of Pt-2 at the age of 57 years. Cerebellar atrophy is evident both in the hemispheres and the vermis. The medulla is mildly atrophic, whereas the pons and midbrain are spared. The anterior-posterior diameter of the spinal cord is shortened from the cervical level to thoracic level, suggesting mild spinal cord atrophy. No intramedullary signal change is found in these atrophic regions.

Supplementary Figure e-2. Representative microphotographs of biopsied nerve specimens

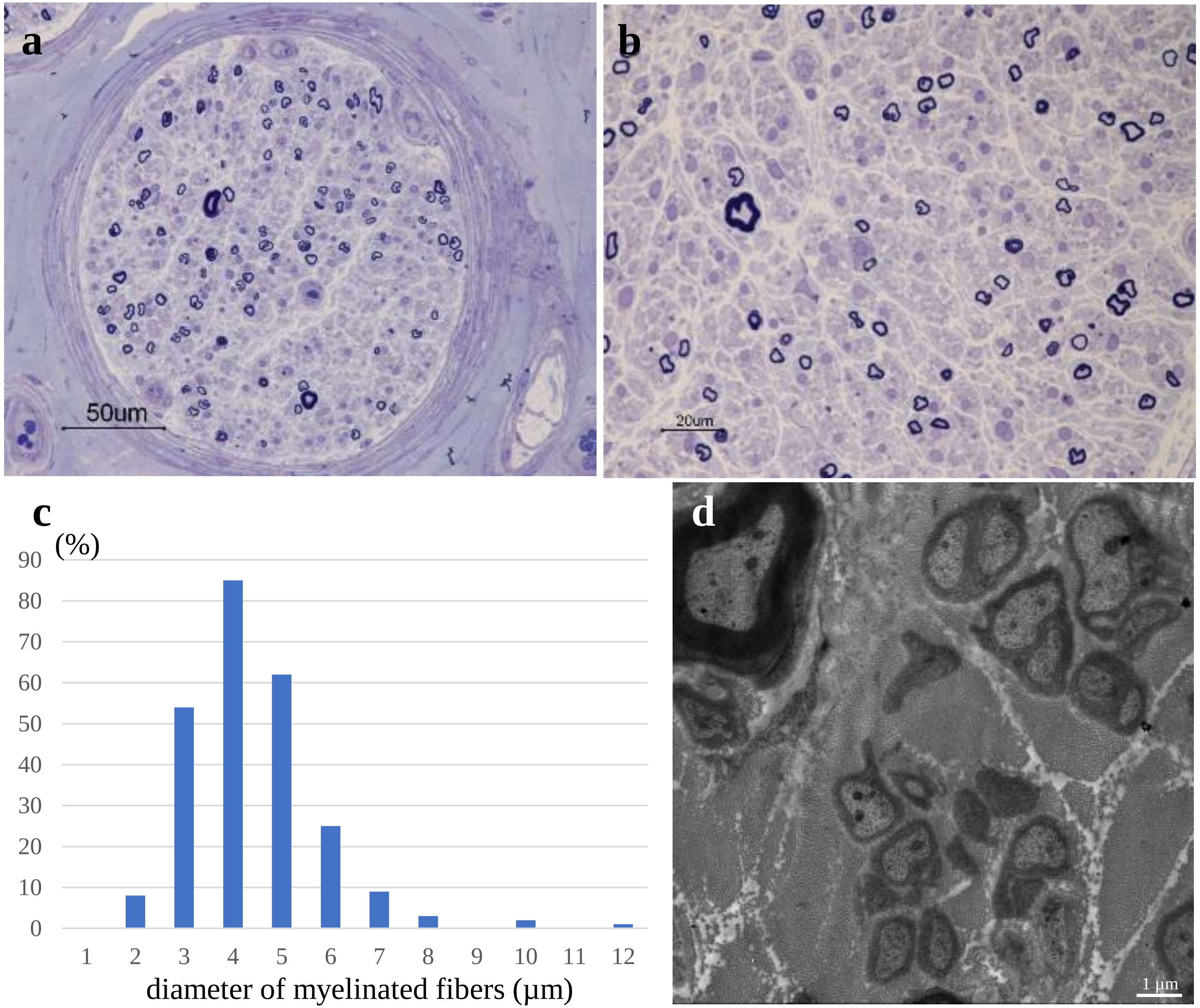

Transverse semi-thin sections of the distal sural nerve obtained from Pt-1 reveal the apparent loss of myelinated nerve fibers with unremarkable regeneration under light microscopy (a, b). There is no inflammation or signs of demyelination. The distributive histogram of the remaining myelinated fibers of Pt-1 indicates preferential large-fiber (>7 μm) reduction (c). The electron micrograph of the sural nerve obtained from Pt-2 at 57 years reveals a normal myelin structure and preserved unmyelinated fibers (d).
