# Supplementary material for: Comprehensive Analysis of a Japanese Pedigree with Biallelic ACAGG Expansions in RFC1 Manifesting Motor Neuronopathy with Painful Muscle Cramps
Source: Cerebellum. 2024 Feb 7;23(4):1498–508. doi: 10.1007/s12311-024-01666-1 (PMC11269323; doi:10.1007/s12311-024-01666-1)
Supplement: Supplementary file 2 — Supplementary file2 (PDF 89 KB) [file 12311_2024_1666_MOESM2_ESM.pdf]

Supplementary Table e-1. PCR primers, conditions for Sanger sequencing and antibodies used for multiple immunohistochemistry

	PCR primers, thermocycling conditions
Nested PCR (1st amplification)	Forward 5'-CCTGAGTCCTCCTGACTGCT-3'<br>Reverse 5'-TCTGTTGAAACCAAGCTCCA-3'<br>98°C 3 min, [98°C 10 s, 62°C (each cycle decreasing by 0.5°C) 15 s, 72°C 3 min] × 10 cycles, [98°C 10 s, 57°C 15 s, 72°C 3 min] × 25 cycles, 72°C 5 min
Nested PCR (2nd amplification)	Forward 5'-GTGGGAGACAGGCCAATCACTTCAG-3'<br>Reverse 5'-TCAAGTGATACTCCAGCTACACCGTTGC-3'<br>98°C 3 min, [98°C 10s, 68°C 15s, 72°C 3 min] × 35 cycles, 72°C 5 min
Probe generation and gDNA template amplification	Forward 5'-CCCCAAAAGCAATTCAGAGA-3'<br>Reverse 5'-CTGCCTTCTCGTGTTGTCCT-3'<br>95°C 4 min, [95°C 30 s, 60°C 30 s, 72°C 60 s] × 38 cycles, 72°C 5 min

Target Protein	Host	Clone/Cat. #	Dilution	Manufacturer
**Primary Antibodies**				
RFC1	Rabbit	GTX129291	1:100	GeneTex
SQSTM1/p62	Mouse	M162-3	1:200	MBL
NCAM/CD56	Mouse	LS-C348390	1:1	LSBio
**Secondary Antibodies/Conjugates**				
Mouse IgG/Alexa Fluor 568	Goat	A-11031	1:400	Invitrogen
Rabbit IgG/Alexa Fluor 488	Goat	A-11034	1:400	Invitrogen
Hoechst33342		H342	1:1000	Dojindo

Supplementary Table e-2. Results of the LTL assay

Individual	Age at blood sampling/sex	*RFC1* expansion	LTL① (kb)	LTL② (kb)	Mean LTL (kb)
Pt-1	62 y M	homozygous	6.49	6.41	6.45
Pt-2	61 y M	homozygous	6.46	6.61	6.54
Pt-3	58 y F	homozygous	7.24	7.12	7.18
II-7	69 y M	heterozygous	7.16	7.08	7.12
II-9	62 y F	no expansion	7.50	7.24	7.37
